# CAFTAN: a tool for fast mapping, and quality assessment of cDNAs

**DOI:** 10.1186/1471-2105-7-473

**Published:** 2006-10-25

**Authors:** Coral del Val, Vladimir Yurjevich Kuryshev, Karl-Heinz Glatting, Peter Ernst, Agnes Hotz-Wagenblatt, Annemarie Poustka, Sandor Suhai, Stefan Wiemann

**Affiliations:** 1 DKFZ, German Cancer Research Center, Division Molecular Biophysics, Im Neuenheimer Feld 580, D-69120 Heidelberg, Germany; 2 DKFZ, German Cancer Research Center, Division of Molecular Genome Analysis, Im Neuenheimer Feld 580, D-69120 Heidelberg, Germany; 3 Dept. Computer Science and Artificial Intelligence, ETSI Informatics University of Granada, C/Daniel Saucedo Aranda s/n 18071, Granada, Spain

## Abstract

**Background:**

The German cDNA Consortium has been cloning full length cDNAs and continued with their exploitation in protein localization experiments and cellular assays. However, the efficient use of large cDNA resources requires the development of strategies that are capable of a speedy selection of truly useful cDNAs from biological and experimental noise. To this end we have developed a new high-throughput analysis tool, CAFTAN, which simplifies these efforts and thus fills the gap between large-scale cDNA collections and their systematic annotation and application in functional genomics.

**Results:**

CAFTAN is built around the mapping of cDNAs to the genome assembly, and the subsequent analysis of their genomic context. It uses sequence features like the presence and type of PolyA signals, inner and flanking repeats, the GC-content, splice site types, etc. All these features are evaluated in individual tests and classify cDNAs according to their sequence quality and likelihood to have been generated from fully processed mRNAs. Additionally, CAFTAN compares the coordinates of mapped cDNAs with the genomic coordinates of reference sets from public available resources (e.g., VEGA, ENSEMBL). This provides detailed information about overlapping exons and the structural classification of cDNAs with respect to the reference set of splice variants.

The evaluation of CAFTAN showed that is able to correctly classify more than 85% of 5950 selected "known protein-coding" VEGA cDNAs as high quality multi- or single-exon. It identified as good 80.6 % of the single exon cDNAs and 85 % of the multiple exon cDNAs.

The program is written in Perl and in a modular way, allowing the adoption of this strategy to other tasks like EST-annotation, or to extend it by adding new classification rules and new organism databases as they become available. We think that it is a very useful program for the annotation and research of unfinished genomes.

**Conclusion:**

CAFTAN is a high-throughput sequence analysis tool, which performs a fast and reliable quality prediction of cDNAs. Several thousands of cDNAs can be analyzed in a short time, giving the curator/scientist a first quick overview about the quality and the already existing annotation of a set of cDNAs. It supports the rejection of low quality cDNAs and helps in the selection of likely novel splice variants, and/or completely novel transcripts for new experiments.

## Background

The availability of a growing number of draft or completed genomes has shifted the most pressing challenges towards the understanding of the molecular and cellular biology of genes and other encoded elements. This understanding is facilitated by the interpretation of genomes not only in terms of their structure but also of their function and diversity. To this end cDNAs from several human large-scale sequencing projects [1-3] have proven their usefulness in the identification and annotation of gene structures and splice forms [4]. These projects have established and improved methodologies for the production of cDNA libraries, enriched in full length and rare transcripts [5-7], which was mandatory for the discovery of new targets for functional genomics. In addition to the utilization of cDNAs in genome and gene annotation, this physical resource provides the basis for the molecular and cellular functional analysis of the encoded proteins [8], and of functional RNAs.

The German cDNA Consortium has pioneered the large-scale sequencing of full-length cDNAs [1] and their systematic exploitation in protein localization experiments [9]. Initial sequence annotations have been integrated with experimental data and bioinformatics analysis [10,8]. One example is the LIFEdb database [11], which is used as a front-end tool for the dissemination of information and for manual and automated annotation. Several large-scale applications of cell-based assays have since been implemented to define candidates for further studies and to identify their potential impact in disease [12], [13]. However, the initial annotation of cDNAs and deduced ESTs has to face a considerable amount of biological and experimental noise [14-16], though some of the observed phenomena (e.g. retained introns) may be biologically significant [17,18]. Accurate cDNA annotation has traditionally been achieved via manual curation, using the experience of expert individuals to annotate sequences manually. Although manual curation can attain high degrees of accuracy [4,19], it cannot keep pace with the continuously growing number of entries in sequence and other databases [20]. A straightforward decision process in the selection of cDNAs for experimental analysis of encoded proteins is therefore a key factor for the creation of relevant datasets within the functional genomics analysis of genes and proteins.

We have developed a new high-throughput cDNA analysis tool, CAFTAN, which filters sequences based on their potential to be derived from full-length and fully processed transcripts and spliced forms. It identifies and filters those cDNAs containing incompletely processed or truncated transcripts, which are consequences of erroneous mRNA processing [16] or of errors in the cloning process. Thus this filtering of targets saves time in the selection of cDNA templates for the subsequent sub-cloning of open reading frames and the functional characterization of encoded proteins.

The main strategy of CAFTAN is based on the mapping of cDNAs to the genome assembly, and the analysis of their genomic context. The presence and type of polyA signals, internal and flanking repeats, the GC-content, and splice site types are evaluated in different tests and aid in the classification of cDNAs into several groups according to their sequence quality. CAFTAN compares the coordinates of cDNAs in the respective genomic locus with the coordinates of a reference set of cDNA-sequences from publicly available resources (e.g., VEGA [21], ENSEMBL [22]). It thus generates detailed information about overlapping exons and a structural classification of any cDNAs with respect to the reference set of transcripts and splice variants.

We applied this tool to a set of 5950 human cDNAs annotated as "known protein-coding" cDNAs in VEGA (Vertebrate Genome Annotation database) [see Additional files 4 and 5] [21]. VEGA is a central repository for manual annotation of vertebrate finished genome sequences. It thus generates detailed information about overlapping exons and a structural classification of any cDNAs with respect to the reference set of transcripts and splice variants. The results showed that CAFTAN was able to classify correctly more than 85% of the analyzed cDNAs. Its good performance makes it suitable for providing the curator/scientist a first and fast overview about the quality and the already existing annotation of a set of cDNA. CAFTAN does not substitute the hand curation process and further detailed ORF analysis; however it supports the selection or rejection of targets, thus speeding up the discovery process in the lab.

## Implemetation

The implementation of the CAFTAN method can be summarized in the following stages: *1) input or raw data, 2) Extraction of simple and composite features from raw data, 3) Rules definitions, 4) Prediction and Evaluation, 5) Program specifications*

### Input (Raw Data)

CAFTAN takes as input a multiple FastA file of the cDNAs to be analyzed and a BLAT output from the FastA sequence file in pSL format with header [23,24].

### Feature extraction

Simple and composite features were extracted from a training set of 3500 manually curated sequences from the German Human cDNA Project -DKFZ collection (supplementary information). Afterwards, the distribution of these features was studied depending on their tag- in the curated DKFZ set- (e.g. "good", "bad", "with deletions", "primed"), see section 2.2.

#### Simple Feature Extraction

##### Exon Mapping Refinement

The first step is to know where and how good the cDNAs are localized in the genome. For that purpose we use BLAT, which maps most cDNAs to a single position/locus in the human genome. However, recent gene duplication events, repeat elements in cDNAs, or misassemble of genome sequence contigs give rise to the mapping of cDNAs at multiple loci, making a post processing of the BLAT output (given by the user) necessary. Only the hit with the lowest number of mismatches is selected. The mapping positions of the selected hit are kept for use of this cDNA in further analyses.

Afterwards, the distances between consecutive blocks- matches of the cDNA to the genome in the BLAT output- are analyzed; a block can be interpreted as an exon. The blocks are joined when the distance is less than 10 bp in the genome sequence. The final number of blocks and their exact genomic positions are recalculated and stored. This process is repeated for every cDNA.

**Figure 1:**
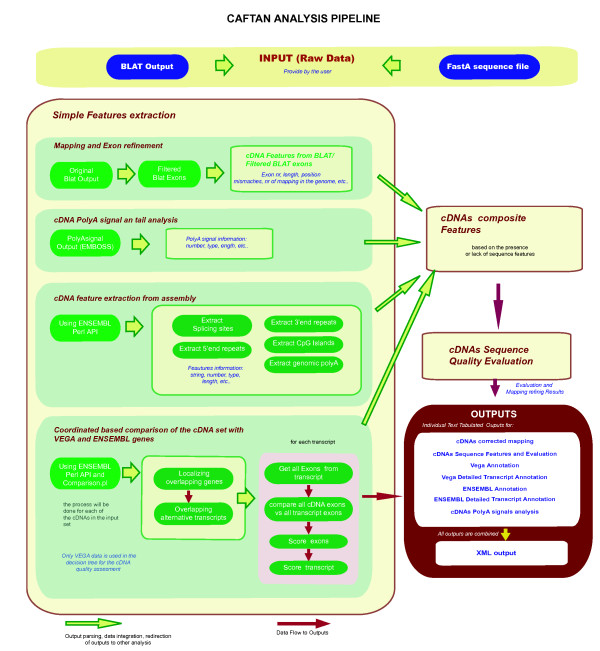
Caftan pipeline, data and program flow.

Additionally information on the number of mismatches, successfully mapped length, number of Ns, gaps etc. are stored for the later generation of composite features and rule definitions. (Figure 1, Table 1).

**Table 1:** cDNA features flow, generation of new features and type of test performed by CAFTAN

**Type**	**Tests**	**Results>**	**CAFTAN input**	**Queries**	**Derived features**
**Mapping**	CDNA coverage	**True **if more than the 20% of the sequence is mapped to the genome			
	3'utr mapping	**True **if delta3 < 50 bp. delta3 is the difference between the length of the cDNA and the last position mapped in the 3' end of the cDNA without poly (A) tail			
	5' utr mapping	**True **if the cDNA start mapping position in the genome is < 28 bp		Filtered BLAT	
	Internal mapping	**True **if deltaint < 50 bp. Delta int is the number of mismatches in the cDNA exons taking into account the cDNA length		Exons	Delta5' region Delta3' region
**Mapping**	Total mapping	**Unmapped: **when 3'utr mapping, 5'utr mapping, and Internal mapping are False		Exons length	Delta int
		**Mapped: **when 3'utr mapping, 5'utr mapping, and Internal mapping are True			
		**Partial mapped**: only two or one of the mapping test are false			

**CDNA Structure**	Single exon cDNA Multiple exon cDNA	**True **if exon number = 1 **True **if exon number > 1	BLAT Output	Filtered BLAT Exons	

**Repeats (R) ***calculated for: 5' upstream, 3' downstream, Last exon*	Repeats number Complex R Repeat type	**Number** **True **if any of the following repeats is present: SVA R, Alu R, L1 R, LTR R, or ScRNA **Returns a string**, the type of repeat in the 3 prima region depending of how many repeats in this region were found and how much can they influence the right cloning of the cDNA: Complex repeats > Simple repeats > Low complexity repeats		Genome assembly query *Repeats overlapping with the given regions*	Simple R Alu R L1 R SVA R LTR R ScRNA R Low complexity R

**Splice Sites (Ss)**	Number of Ss cDNA Ss type Ss-score	**Number** **Returns a string **with the type depending on the Ss types in the cDNA **Unknown: **at least one Unknown splice sites **Antisense**: at least one antisense Ss, no Unknown **U12**: at least one U12 Ss, no antisense and no unknown Ss **Non_canonical**: only non canonical and cannonical Ss **Canonical: **All splice sites are cannonical **Percent. **Returns the % of good splice sites in a multi exon cDNA. Good splice sites are canonical, non canonical and u12 splice sites		Genome assembly query	Canonical Ss "GT-AG" Non_canonical Ss "GC-AG" U12 Ss "AT-AC" Antisense Ss "CT-GC","GT-AT" Unknown Ss (others)
		**Returns a string **with the type depending on the Ss types in the cDNA			
	cDNA Ss type	**Number** **Unknown: **at least one Unknown splice sites			Canonical Ss "GT-AG"
		**Antisense**: at least one antisense Ss, no Unknown			Non_canonical Ss "GC-AG"
		**U12**: at least one U12 Ss, no antisense and no unknown Ss		Genome assembly query	U12 Ss "AT-AC"
		**Non_canonical**: only non canonical and cannonical Ss			Antisense Ss "CT-GC" "GT-AT"
	Ss-score	**Canonical: **All splice sites are cannonical			
		**Percent. **Returns the % of good splice sites in a multi exon cDNA. Good splice sites are canonical, non canonical and u12 splice sites			Unknown Ss (others)

**PolyA signal and tail**	cDNA signal type Poly A tail	**Returns a string **with the type depending on: **Canonical: **There is at least a canonical signal in the cDNA **Non-canonical a**: There is no canonical signal and there is a non-canonical a onel **Non-canonical b**: There is none of the above signals but there is a non-canonical b one **Non-canonical c: **There is none of the above signals but there is a non-canonical c one **True **if there is a poly (A) tail in the cDNA	Polyasignal Output		*Signals:* Canonical A [TA]TAAA Non-canonical a [^A]ATAAA Non-canonical b AATA[^A]A Non-canonical c A[CG]TAAA Tail length
**Contamination**	Contamination	**Returns a string **with the type of contaminations **PolyA C: **if there is more than 80% As in a 20 bp window in the genome. 5 last bp from the last exon + 15 bp after. **RepeatC: **Complex repeats contamination in the last exon or 3' end **Mixed contamination: **both contaminations **No Contamination: **no polyA C and no RepeatC	Polyasignal Output	Genome assembly query	Genomic polyA tail Presence of complex Repeats

##### cDNA polyA signal and tail analysis

PolyA sites in mRNAs are defined by a hexameric polyadenylation signal, AAUAAA, or a one-base variant thereof [25]. This signal is usually located ~15 bases upstream of the cleavage site and, sometimes, there is also a GU (Guanosyl Uridine)-rich element located 20–40 bases downstream of the site [26]. The polyA polymerase then adds a polyA tail to mRNAs during pre-mRNA maturation.

We have developed the program "Polyasignal", using EMBOSS libraries [see Additional files 2 and 3] [27], to identify within cDNA sequences potential polyA signals in the context of a polyA tail. The program retrieves the position and type of every possible hit. It also allows searching for sequences having only polyA signals or polyA tail.

The user can define almost all variables, such as maximum distance allowed between the signal and the polyadenylation site (default 50nt), and patterns to match the polyA signals and polyA tail. Parameters are by default optimized for the human genome [25,28] but it can be easily adapted for other organisms. All obtained information is stored for the later creation of composite features and rules extraction.

##### Feature extraction from assembly: splice sites, repeats, genomic polyA tail

###### Splice sites

Most exon/intron boundaries match consensus dinucleotides that are specific for splice donor and splice acceptor sites at the 5' and 3' ends of exons, respectively [29]. All putative splice donor and acceptor sites (Ss) are extracted for every cDNA from the genome assembly (Table 1). U2 dependent introns have been historically classified in those containing canonical splice sites with conserved GT and AG dinucleotides and those containing GC-AG were called non-canonical splice sites. U12-dependent introns discovered later showed a preference for AT-AC dinucleotides in the splice donor/acceptor positions. We use this classification to create the introns Ss types (Table 1: canonical, non-canonical, u12 and unknown Ss), because there is not an accepted standard nomenclature in the literature for the splice sites depending on the splicesome, or on the combination of donor/acceptor dinucleotides used by the intron.

Once the splice type of each intron in a cDNA has been defined, the Ss type of the whole cDNA is then defined (Table 1). The cDNA *Splice Site Type *is classified as canonical only when *all *of its splice sites are canonical, for other cases see Table 1.

###### CpG islands

The genomic sequence is further checked for CpG islands up to 200 bp from the beginning of the cDNA. We use the generally accepted definition of what constitutes a CpG island [30].

###### Genomic polyA tail

Additionally, the presence of a stretch of multiple A's (polyA tail) in the genome at the matching position of the cDNA's end is checked. We look for a window of 20 bp in the genome taking the last 5 bp of the last exon plus the following 15 bp in the genome. If this 20 bp nucleotide window has more than 80% A's content, then we assume the presence of a genomic polyA.

###### Repeats

Repeat elements in the last exon and in the 5' and 3'end of thequery cDNA are identified. The repeats in these regions are searched as follow: those overlapping the last block/exon of a cDNA, those overlapping the first 50 bp in the 5'upstream region, and repeats overlapping the terminal 50 bp from the 3'downstream region. All information is stored for the later generation of composite features, rules and quality tests evaluation (Figure 1)

### Coordinate based comparison of the cDNA set with VEGA and ENSEMBL annotations

CAFTAN compares the coordinates of mapped cDNAs after filtering with the genomic coordinates of reference sets from the publicly available resources of the Vertebrate Genome Annotation (VEGA) database [21] and ENSEMBL [22](Figure 1). VEGA is a central repository for manual annotation of vertebrate finished genome sequences while ENSEMBL produces and maintains automatic annotation on many eukaryotic genomes.

Each analyzed cDNA is linked to the best matching gene in VEGA and/or ENSEMBL (Figure 1). Since VEGA genes are organized in transcripts, the best matching transcript needs to be selected. For that purpose, the coordinates of all existing VEGA and/or ENSEMBL transcript variants for the best matching gene are extracted and compared with the query cDNA including all exon and intron coordinates. An "*exon score*" defined as the ratio between the exons overlap and the sum of their lengths, is calculated for all transcript exons and query cDNA exons. Once the exons have been compared all to all a "*transcript score*" *given *below is calculated for every VEGA and/or ENSEMBL transcript found to overlap with the query cDNA.

Transcript score = ((2*total_transcript_exon score)/(mrna_exon_nr + cdna_ex_nr));

Here the "*total_transcript_exon*" score is the sum of all transcript exon scores and "*mrna_exon_nr*" and "*cdna_ex_nr*" are the number of exons in the transcript and in the query cDNA, respectively. The score can have values between 0 and 1, where 1 indicates a perfect match in all exons from the VEGA/ENSEMBL transcript to all the exons of the query cDNA and the same number of exons. The transcript with the highest score is selected as the best overlapping one to the query cDNA. All its relevant information is stored.

#### Composite Features

The extracted features are combined to calculate further relevant information for each cDNA sequences (Figure 1, composite features in Table 1).

##### Mapping

First the mapping of a cDNA sequence to the genome is analyzed in detail. The percentage of a cDNA sequence that can be mapped to the genome gives an indication of possible colligation events during cDNA cloning, potential errors in the genome assembly, or the existence of trans-splicing. All information obtained from the raw BLAT output and its filtering is used to calculate the quality of the mapping process by taking into account the number of mismatched positions internally and in the 3' and 5' ends (Table 1).

##### cDNA Structure

The tests related to the cDNA structure determine if the query cDNA was the product of a single exon or a multi-exon gene and is calculated using the filtered BLAT Exon results.

###### Repeats

the number of repeats in the three regions described before in section 2.1.3, is analysed and post-processed to distinguish between simple repeats, low complexity repeats (mono-, di-, and tri-nucleotide repeats), and complex repeats (SVA, Alu, LINE, SINE, LTR, ScRNA, other complex repeats). The repeat type composite feature in the corresponding region depends on how many repeats were found and how much they could influence the right cloning of the cDNA: Complex repeats > Simple repeats > Low complexity repeats.

###### Splice sites score

Once the splice type of each intron in a cDNA has been defined, the Ss type of the whole cDNA is then defined (Table 1). The Ss-score is calculated as the percentage of "good" splice sites in a multi-exon cDNA. Splice sites are rated "good" when they match canonical, non-canonical, or u12 donor-acceptor pairs. A higher Ss-score correlates with a better quality of the respective cDNA. In the training set, cDNAs sequences with internal deletion, when compared to a reference sequence, presented always a variable percentage of bad splice sites along the total number of splice sites. Therefore, the presence and type of splice sites was valued as a positive feature for the cDNA classification within CAFTAN. Such deletions are commonly artefacts that occur in the bacteria after cloning, where sequences of cDNA inserts are deleted by recombination. Since this recombination is independent of splicing, these events mostly do not occur at canonical splice sites

###### polyA signal and tail

The number and type of signals are calculated for each cDNA using PolyAsignal and the results are used to create the feature signal type (Table 1). The signal type is selected according to the hexamer distribution [25] found. The polyA signal selection ranking is in the following order, in cases where more than one signal is found in one cDNA: canonical > non-canonical_a > non-canonical_b > non-canonical_c. Definitions of the hexamer types are given in Table 1.

###### Contaminations

A polyA contamination is true if there is a polyA stretch in the genome at the 3' mapping position of the terminal cDNA exon, this is classified as a genomic polyA. Then the 3' end of the last exon is checked for the presence of complex and simple repeats within its last 50 bp or 20 bp respectively. If repeats are found in these regions the cDNA has "repeat contamination". If more than one repeat is found, only one will be shown with the following preference: First complex repeats in the following order, Alu > L1 > SVA > LTR, then simple repeats and last low complexity repeats.

"Mixed contamination" means both types, ie. genomic polyA and repeat contamination. The type of any putative contamination is classified and stored (Table 1).

## 3. Rule generation

The distribution of simple and composite features extracted from the training set (3500 DKFZ manually curated cDNAs) was studied depending on their tag- in the curated DKFZ set- (e.g. "good", "bad", "with deletions", "primed") using clustering methods. We found distinct relationships among features that were able to characterize different sets of observations in the training set. These profiles -relationships among features- were discussed and some times modified according to the curator's expert knowledge. The decision rules obtained are presented in Table 2, and use the terminology from Table 1.

**Table 2:** Decision rules for cDNA sequence quality evaluation in CAFTAN.

**Type**	**Coverage**	**Mapping**	**Exons**	**Sscore (%)**	**SS_type**	**Contamination_test**	**Signal**	**Tail**	**VEGA match**
**Uncovered**	False								
**Bad_se_cdna**	True	Unmapped	1						
**Bad_me_cdna**	True	Unmapped	> 1						
**Bad_se_cdna**	True	Partial mapped	1						
**Bad_me_cdna**	True	Partial mapped	> 1	< 100					
**Questionable_me**	True	Partial mapped	> 1	100		No contamination			
**Bad_me_cdna**	True	Partial mapped	> 1	100		Any contamination			
**Good_se_cdna**	True	Mapped	1			No contamination	+	+	
**Bad_se_cdna**	True	Mapped	1			No contamination	-	-	
**Questionable_se**	True	Mapped	1			Genomic polyA	+	+	Perfect
**Bad_se**	True	Mapped	1			Genomic polyA	+	+	No Perfect
**Bad_se_polyA_primed**	True	Mapped	1			Genomic polyA	+	-	
**Good_se**	True	Mapped	1			No contamination	+	-	Perfect
**Questionable_se**	True	Mapped	1			No contamination	+	-	No Perfect
**Bad_se**	True	Mapped	1			Repeats	-	-	
**Bad_se_polyA_primed**	True	Mapped	1			No contamination	-	+	
**Good_se**	True	Mapped	1			Repeats	+	-	Perfect
**Questionable_se**	True	Mapped	1			Repeats	+	-	No Perfect
**Bad_se**	True	Mapped	1			Genomic polyA	-	-	
**Bad_me**	True	Mapped	> 1	Ssc <= 60					
**Bad_me**	True	Mapped	> 1	60 < Ssc < = 100	Good SS*	Mixed contamination			
**Bad_me**	True	Mapped	> 1	60 < Ssc < 80	Good SS*	Genomic polyA			
**Bad_me**	True	Mapped	> 1	Ssc = > 90	Bad SS +	Mixed			
**Bad_me**	True	Mapped	> 1	60 < Ssc < 90	Bad SS +				
**Good_me_cdna**	True	Mapped	> 1	60 < Ssc <= 100	Good SS*	No contamination			
**Good_me_cdna**	True	Mapped	> 1	60 < Ssc <= 100	Good SS*	No contamination	+	+	
**Good_me_cdna**	True	Mapped	> 1	60 < Ssc <= 100	Good SS*	No contamination	+	-	
**Good_me_cdna**	True	Mapped	> 1	Ssc > = 80	Good SS*	No contamination	-	-	
**Good_me_cdna**	True	Mapped	> 1	Ssc > = 80	Good SS*	No contamination	-	+	
**Good_me_cdna**	True	Mapped	>1	Ssc > = 80	Good SS*	Repeats			
**Good_me_cdna**	True	Mapped	> 1	Ssc > = 90	Bad SS +	No contamination			
**Good_me_cdna**	True	Mapped	> 1	Ssc > = 90	Bad SS +	Repeats			
**Questionable_me**	True	Mapped	> 1	60 < Ssc < 80	Good SS*	No contamination	-	-	
**Questionable_me**	True	Mapped	> 1	60 < Ssc < 80	Good SS*	No contamination	-	+	
**Questionable_me**	True	Mapped	> 1	Ssc > = 80	Good SS*	Genomic polyA			
**Questionable_me**	True	Mapped	> 1	60 < Ssc < 80	Good SS*	Repeats			
**Questionable_me**	True	Mapped	> 1	Ssc > = 90	Bad SS +	Genomic PolyA			

The definition of splice site types (Ss type) is described in Table 1. * Good SS are Canonical Splice sites, non-canonical, and u12. Bad SS + are unknown and antisense combinations of donor and acceptors (Table 1)

1.) "Uncovered", i.e. those cDNAs that mapped with less than 20 % of their length to the genome, which usually means the presence of too many gaps in the genome 2.) "Bad_se", i.e. bad single exon cDNAs; 3.) "Bad_me", i.e. bad multiple exon cDNAs; 4.) "Questionable_se", and 5.) "Questionable_me", are cDNAs with single or multiple exons, respectively, that should be manually inspected by the curator/scientist in order to take a decision about their quality; 6.) "Good_se_cdna", i.e., high quality single exon cDNAs; 7.) "Good_me_cdna", i.e., high quality multiple exon cDNAs; 8) "Partial_mapped", are cDNAs with gaps bigger than 20 bp either in the 3', 5' end or in the internal region 9.) "Undefined", are any cDNAs that do not fit into one of the previous categories because they failed to pass the evaluation pipeline. Elements in the latter group will be used for the further refinement of the evaluation and classification system.

The quality assessment starts with the evaluation of the quality of a cDNA mapping to the genome. The decision process is similar for multiple and single exon cDNAs (Table 2). However, for the former ones further information is required i.e., the Splice Sites Type definition (Ss type) and especially the Splice Sites Score. The Splice Sites Score (Table 1) was defined as the percentage of canonical, non-canonical and u12 donor-acceptors pairs in the cDNA across the total number of donor-acceptor pairs. A cDNA is not allowed to contain antisense other unknown Ss to be classified as "Good splice sites". In contrast, the "Bad" Splice Sites Type is attached to a cDNA when there is at least one unknown or antisense splice site among the donor acceptor pairs of a cDNA.

A cDNA is rated to be of high quality when it is perfectly mapped to the genome, has a polyA signal and a polyA tail, and in the case of multiple exons the Ss score is higher than 60 % and the Ss type is "Good splice sites" (Table 2). The Ss score is required to be higher than 90 % for a cDNA, to be classified as high quality, in the case that it contains "Bad splice sites". This means that at least one of the donor-aceptor pairs is unknown or antisense. cDNAs lacking the polyA signal are considered to be "Bad". Any multiple exon cDNAs having Ss scores lower 60% are classified as "Bad_me" (refer to section 2.2.4).

cDNAs with one type of contamination repeats, or genomic polyA and/or lacking the polyA tail need to fulfill other criteria in order not to be classified as "Bad" (Table 2). The presence of complex repeats in the last exon of the cDNA is considered negatively in the quality selection process. Alu sequences, like other SINEs, occur at higher frequency within non-coding domains of single copy genes (e.g. inter-genic regions, introns, 5' and 3' UTRs, etc) and they can affect the processing of pre-mRNAs leading to altered gene products [31]. In addition, the presence of Alu sequences and other retro-transposable elements (Mir, LINES, ect) in pre-mRNA can affect Polyadenylation of transcripts and influence transcription as well [32,33]. These repeats can be present in total RNA pool used to obtain cDNA library (e.g., when RNA polyA enrichment fails). This situation makes possible the priming of other mRNAs -especially partially spliced- during amplification (DKFZ curated data). As result incomplete/problematic cDNA sequences are produced. (Intronic) Alu repeats frequently contain polyA sequences at the genomic level and these often serve as oligo dT-priming sites in the cDNA generation process. Such cDNAs are usually 3' truncated and mostly terminate in intronic sequences. Since this phenomenon is quite frequent we included the presence of an Alu sequence at the 3' terminal end of a cDNA as "bad". But, as observed in Table 2, if a cDNA has a complex repeat in the last exon but all other composite features are following the profile of a good cDNA, it will classified as "questionable_cdna".

The lack of a polyA tail at the 3' end is also negatively considered during the quality assessment process, because a polyA tail is a canonical motif that strongly promotes translation initiation through secondary structures, such as hairpins [34]. And moreover polyA+ RNAs have been found in the interchromatin granule clusters (IGCs) in the nucleus which are not transported to the cytoplasm, as would be the case if they represented protein-coding mRNAs, but do have regulatory functions [35].

CAFTAN uses the information recovered from VEGA and ENSEMBL (Table 2). If there is a perfect match of a VEGA/ENSEMBL transcript ("*best_transcript score*" > 0.98) to the query cDNA, then the cDNA will be classified as "Good" or "Questionable". If the match is not perfect ("*best_transcript score*" <= 0.98) the classification will depend on the presence/absence/type of contamination and/or the presence or absence of polyA signal and polyA tail (for details look at Table 2). If there is at the same time a mixed contamination (Genomic polyA and repeats contamination) the cDNA will be considered always "Bad".

Although the presence of CpG islands is always checked, their presence or absence is not taken into account in the decision tree. It has been proven in mammalian genomes that many genes are not necessarily associated with these regions. Nevertheless, this feature could be useful in the detection of 5' cDNA's completeness by further manual verification. A major amount of protein-coding genes transcription start sites (TSS) – around 70% – are found to be near CpG islands [36].

### Evaluation sets

We generated and analyzed two cDNA sets in order to evaluate the performance of our method. The first set contained 5,954 "known protein-coding" cDNAs extracted from the human VEGA database, which is the current "golden" annotation standard in mammalians. For comparison we created a set of 3,000 "bad" cDNAs sequences. To do this, we selected from the human genome i) randomly a chromosome, ii) the beginning of the cDNA according to the length of the randomly selected chromosome, and iii) a random number of exons, introns and their lengths. Limits for the introns and exons lengths were taken from the work of Sakharkar et al., [37]. Randomly generated exons were retrieved from the human assembly NCBI36 and joined to generate cDNAs. For this set of "bad" cDNAs a polyA tail was artificially added at the 3'end of the terminal "exon".

## Program specifications

CAFTAN was written using Object Oriented Perl, and has been implemented as a downloadable stand-alone version available at [38]. Preprocessing of cDNA sequences with the program Polyasignal is mandatory for the stand-alone version of CAFTAN, as this takes output files of Polyasignal as input. CAFTAN requires a previous installation of Bioperl-1.2 (or greater) and of ENSEMBL [22] because it utilizes the ENSEMBL Perl API [39] and EMBOSS (up to version 3.0).

CAFTAN is also available under the W3H task system at the DKFZ [40]. This Web version takes as input multiple Fast A sequences file and a BLAT [23] output from the FastA sequence file in pSL format with header.

The Polyasignal source code is available through the authors and it has been submitted to be included in future releases of the EMBOSS package. Queries in the ENSEMBL database can be done "remote" at the ENSEMBL site or "locally" provided the user has installed the ENSEMBL databases. The default is set to remote.

## Results and Discussion

CAFTAN allows the analysis of several thousands of cDNAs within a few minutes, giving the curator/scientist an overview about the likelihood of having fully spliced/processed cDNAs and providing the already existing annotation for the cDNA set. It supports the rejection of low quality cDNAs and helps selecting novel splice variants, and/or completely novel transcripts for new experiments.

The evaluation described in the implementation section to assess the performance of CAFTAN showed that it is able to correctly classify more than 85% of 5950 selected "known protein-coding" VEGA cDNAs as high quality multi- or single-exon (Table 3). It identified as good 80,6 % of the single exon cDNAs and 85 % of the multiple exon cDNAs. In the set of random generated cDNAs the fraction of cDNAs that was predicted, as high quality was only 5 %, almost all being single exon cDNAs. VEGA is a central repository for manual annotation of vertebrate finished genome sequences thus the "known protein coding" cDNA set was selected as a reference for the evaluation.

**Table 3:** CAFTAN results for the annotated VEGA cDNAs and for the 3000 randomly generated cDNAs.

**CDNA**	**Known VEGA Transcripts**		**Random Sequences**	
	
	**Number**	**Percent**	**Number**	**Percent**
**Good single exon cDNA**	154	2.58 %	46	1.53 %
**Good multiple exon cDNA**	4911	82.53 %	1	0.03 %
**Questionable single exon cDNA**	0	0.00 %	0	0 %
**Questionable multiple exon cDNA**	57	0.96 %	0	0 %
**Bad single exon cDNA**	37	0.62 %	115	3.83 %
**Bad multiple exon cDNA**	791	13.29 %	2666	88.86 %
**Not mapped**	0	0.00 %	172	5.73 %
**Total**	5950		3000	

When we analyzed the VEGA cDNAs classified by CAFTAN as "Bad_se" (37/191) and "Bad_multiple_exons" (791/5759) we found, that in the case of the group classified as "bad_multiple_exon_cdna", all of them lacked the presence of a polyA signal but had a perfect match to VEGA transcripts. The manual inspection by an expert curator of these VEGA CDNAs sequences showed that most of the VEGA cDNAs sequences were not complete, part of the 3'UTR region failing or having an artefact 3'UTR end. In Table 4, there is a summary of 15 selected cases, in which only 2 of these cases (No. 4 and No. 8) are false negatives, (e.g. case No. 8 has a non common polyA signal which is not contemplated in the Polyasignal program). On the other hand, when inspecting the VEGA cDNAs group classified as bad single exon ("Bad_se" 37/191) we found that most of them lacked a polyA signal or had a non-conventional one. When checked in detail (Table 5), 19 of these 37 "bad_single_exons" were found to be Histone coding cDNAs. Histone coding cDNAs do not undergo polyadenylation due to the fact that they have a very short life and do not need to be stabilized. The rest either did not have a complete 3'UTR or they were even products of internal priming events [41,42] complicating a proper distinction between these cases. While the lack of a polyA signal does not necessarily indicate an artifact, internal priming certainly is, and such cDNAs must be regarded as noise for our porpouse. Taking these results into account, the CAFTAN can correctly classify up to 90 % of the cases, thus proving to be highly efficient. The number of "right" and "wrong" classified single exon cDNAs in the VEGA set was very similar to the respective values in the random set.

**Table 4:** Selection of cDNAs classified as "bad_multiple_exon_cdna" by CAFTAN.

	**cDNA_Name**	**Signal**	**VEGA_matcht**	**VEGA match**	**Problem**
1	**OTTHUMT00000002088**	No signal	perfect	TNFRSF14-001	The sequence ends with the poly A signal AAUAUA
2	**OTTHUMT00000002210**	No signal	perfect	SLC35E2-001	Partial Sequence in the 3' UTR end. The sequence is longer and for that reason is not possible to find the polyA signal
3	**OTTHUMT00000003581**	No signal	perfect	PARK7-005	Partial Sequence in the 3' UTR end. The sequence is longer and for that reason is not possible to find the polyA
4	**OTTHUMT00000003582**	No signal	perfect	PARK7-006	RPL20 Gen, eventually (A)ATGAA(A) signal
5	**OTTHUMT00000004064**	No signal	perfect	C1orf86-001	Not a perfect cDNA, the last exon fails and for that reason it is not possible to find a polyA signal
6	**OTTHUMT00000004201**	No signal	perfect	PHF13-001	False 3' end (artefact), the real 3' end is upstream from this point and is supported by many ESTs and a canonical polyA signal
7	**OTTHUMT00000005013**	No signal	perfect	CTNNBIP1-002	Good cDNA, this cDNA does not have canonical or typical non-canocial polyA signals checked in CAFTAN. Putative polyA signal: ATGTAAATAT
8	**OTTHUMT00000005014**	No signal	perfect	CTNNBIP1-003	The real 3' UTR is a little bit longer and contains a canonical polyA signal 20 bp upstream from the polyA tail.
9	**OTTHUMT00000005015**	No signal	perfect	CTNNBIP1-004	Partial Sequence in the 3' UTR end, fail the terminal bases, which make a perfect canonical polyA signal (AAUAAA) 15 bp upstream from the polyA tail
10	**OTTHUMT00000005017**	No signal	perfect	UBE4B-002	Partial Sequence in the 3' UTR end, fail many terminal bases, which make a perfect canonical polyA signal (AAUAAA)
11	**OTTHUMT00000005070**	No signal	perfect	SDF4-002	Partial Sequence in the 3' UTR end, fail almost all the 3 'UTR
12	**OTTHUMT00000005103**	No signal	perfect	KIF1B-002	Partial Sequence in the 3' UTR end, fail almost all the 3 'UTR
13	**OTTHUMT00000005105**	No signal	perfect	KIF1B-004	Partial Sequence in the 3' UTR end, fail almost all the 3 'UTR
14	**OTTHUMT00000005430**	No signal	perfect	UBE2J2-001	Partial Sequence in the 3' UTR end, fail almost all the 3 'UTR

All of them lack the presence of a polyA signal but have a perfect match to VEGA transcripts, as it should be expected. The manual inspection by an expert curator of these VEGA CDNAs showed that all of them but 2 (No. 4 and No. 8) where not complete failing part of the 3'UTR region or having artifact 3' end like No. 6.

**Table 5:** Selection of cDNAs classified as "bad_single_exon_cdna" by CAFTAN.

	*VEGA Transcript ID*	***Description***	**Transcript name**	***Problem***
**1**	**OTTHUMT00000033440**	histone 2, H2ab	HIST2H2AB-001	Histone mRNAs are not polyadenylated.
**2**	**OTTHUMT00000040062**	Small proline-rich protein 1A	SPRR1A-001	Fails tail of the 3' end of the cDNA
**3**	**OTTHUMT00000040078**	histone 1, H3c	HIST1H3C-001	Histone mRNAs are not polyadenylated.
**4**	**OTTHUMT00000040083**	histone 1, H2bb	HIST1H2BB-001	Histone mRNAs are not polyadenylated.
**5**	**OTTHUMT00000040098**	histone 1, H3f	HIST1H3F-001	Histone mRNAs are not polyadenylated.
**6**	**OTTHUMT00000040100**	histone 1, H2ad	HIST1H2AD-001	Histone mRNAs are not polyadenylated.
**7**	**OTTHUMT00000040108**	histone 1, H2bf	HIST1H2BF-001	Histone mRNAs are not polyadenylated.
**8**	**OTTHUMT00000040109**	histone 1, H2bg	HIST1H2BG-001	Histone mRNAs are not polyadenylated.
**9**	**OTTHUMT00000040110**	histone 1, H2bh	HIST1H2BH-001	Histone mRNAs are not polyadenylated.
**10**	**OTTHUMT00000040111**	histone 1, H2bi	HIST1H2BI-001	Histone mRNAs are not polyadenylated.
**11**	**OTTHUMT00000040119**	histone 1, H4h	HIST1H4H-001	Histone mRNAs are not polyadenylated.
**12**	**OTTHUMT00000040138**	histone 1, H2	HIST1H2BJ-001	Histone mRNAs are not polyadenylated.
**13**	**OTTHUMT00000040154**	histone 1, H2aj	HIST1H2AJ-001	Histone mRNAs are not polyadenylated.
**14**	**OTTHUMT00000040160**	histone 1, H2al	HIST1H2AL-001	Histone mRNAs are not polyadenylated.
**15**	**OTTHUMT00000040162**	histone 1, H2am	HIST1H2AM-001	Histone mRNAs are not polyadenylated.
**16**	**OTTHUMT00000042255**		RP11-295F4.3-001	Fails tail of the 3' end of the cDNA
**17**	**OTTHUMT00000042262**		TAAR8-001	Signal 70 pb upstream. EST evidence from a shorter gene
**18**	**OTTHUMT00000043372**	histone 1, H1c	HIST1H1C-001	Histone mRNAs are not polyadenylated.
**19**	**OTTHUMT00000043452**	histone 1, H3i	HIST1H3I-001	Histone mRNAs are not polyadenylated.
**20**	**OTTHUMT00000043453**	histone 1, H3j	HIST1H3J-001	Histone mRNAs are not polyadenylated.
**21**	**OTTHUMT00000043884**	histone H1	HIST1H1A-002	Histone mRNAs are not polyadenylated.
**22**	**OTTHUMT00000044894**	cysteinyl leukotriene receptor 2	CYSLTR2-001	Partial 3'UTR Sequence
**23**	**OTTHUMT00000050610**	G protein-coupled receptor 10	GPR10-001	Partial 3'UTR Sequence
**24**	**OTTHUMT00000051889**	Interferon, alpha 4	IFNA4-001	Internal primed
**25**	**OTTHUMT00000051905**	Interferon, alpha 6	IFNA6-001	Internal primed
**26**	**OTTHUMT00000053504**	T-cell acute lymphocytic leukemia 2	TAL2	Internal primed
**27**	**OTTHUMT00000057327**	G protein-coupled receptor 174	GPR174	Partial 3'UTR Sequence
**28**	**OTTHUMT00000057879**	Insulin receptor substrate 4	IRS4	Ends in the signal,
**29**	**OTTHUMT00000059081**	Potassium voltage-gated channel	KCNA10-001	Partial 3'UTR Sequence
**30**	**OTTHUMT00000059124**	Taste receptor, type 2, member 4	TAS2R4	Partial 3'UTR Sequence
**31**	**OTTHUMT00000072293**	A disintegrin	ADAM21-001	Artifact, much longer than the real gene
**32**	**OTTHUMT00000076126**	MAS1 oncogene-like	MAS1L-001	Partial 3'UTR Sequence
**33**	**OTTHUMT00000087128**	histone 2, H2ac	HIST2H2AC-001	Histone mRNAs are not polyadenylated.
**34**	**OTTHUMT00000147346**		HsG1428-001	Partial 3'UTR Sequence
**35**	**OTTHUMT00000147982**		HsG647-001	Partial 3'UTR Sequence
**36**	**OTTHUMT00000147994**		HsG684-001	Presence of a repeat in the 3' UTR
**37**	**OTTHUMT000001480 95**		HsG2001-001	Partial 3'UTR Sequence

All of them lack the presence of a polyA signal but have a perfect match to VEGA transcripts, as it should be expected. The manual inspection by an expert curator of these VEGA CDNAs showed that 19 all of them are Histone coding cDNAs which do not go under polyadenylation. The rest either did not have a complete 3'UTR region or was an artifact 3'.

It is worth mentioning that the *"Splice Sites Score" *is the composite features that provided the most reliable information about the quality of multi-exon cDNAs.

When analyzing the remaining "bad" multiple exons (false negatives) cDNAs, we saw that most of the wrong classification was due to mistakes in the mapping process. BLAT is the current fastest algorithm to map sequences to a genome. It is designed to find sequences of 95% and greater similarity in a minimum of 33 bases, thus it may miss shorter sequence alignments, making the mapping of shorter exons especially problematic. An alternative would be the use of MEGABLAST [43], and the subsequent use of SIM4 [44]. We have already used such an approach for all mammalian genomes in cDNA2Genome [45] and recently the same approach was used for *Arabidopsis thaliana *by Hayden *et, al*[46]. This approach implies a much larger processing time and a big bias through the selection of exons containing canonical splice sites by Sim4 [47]. Other programs like EST_Genome [48] or like Spidey [49] are also accompanied by strong preferences in it's scoring for GT...AG splicing sites [50]. This bias is reduced when using BLAT because it provides pair-wise alignment information but not explicit predictions of splice junctions.

Several cDNAs were wrongly classified because of misalignments in the first and last exons that were due to the presence of repeat elements in the UTR regions, and to misalignments of 3' terminal poly-A sequences. All these cDNAs were classified as being of questionable quality. Any cDNAs in this category should not be discarded but a rating in this category should rather be an indicator to the scientist curator that he or she should look in detail in the CAFTAN output and to take an expert decision on the quality of the query cDNA. The cDNAs in this category were about 5% in the multi-exon class of cDNAs.

The transcript score, which evaluates the total overlap between a query cDNA and the VEGA/ENSEMBL annotated transcripts, was for both cDNAs types -when using the human NCBI assembly 35- between 1 and 0.8 in 99.5 % of the cases, where 1 is a perfect match. The results presented here, are based on assembly 36 and so when looking in the VEGA annotation, the average of the transcript score is lower. This is due to the fact that VEGA still uses the assembly NCBI35, while ENSEMBL and all the databases available for BLAT use the assembly NCBI36. This is a temporal problem that affects only to those chromosomes, which changed the mapping coordinates in the NCBI36 and will be solved in the moment that VEGA changes to the assembly NCBI36. In the random generated cDNAs sets all transcript scores remained under 0.23 %.

CAFTAN was implemented as a fast approach towards the selection of cDNAs from resources that have been generated in high-throughput projects. A more accurate algorithm like MegaBlast would certainly improve the alignments, but this would be at the cost of speed. In view of the high success rate of CAFTAN in the analysis of the VEGA and random cDNA sets already obtained with BLAT, we decided not to compromise speed for a possible small improvement in reliability. Nevertheless, future implementations of CAFTAN will be allowing users to upload either BLAT or MegaBlast outputs or they can already use cDNA2Genome, which annotates cDNAs using this approach but which needs long running times. Both the WEB and the downloadable versions are modular and will further permit the implementation of additional filtering tools for alternative mapping methods.

Additionally to the cDNA quality classification, CAFTAN provides extensive information about each cDNA, which is divided in different tab tabulated outputs tables (Figure 1). The "*Mapping*" output contains the exact mapping information of the cDNAs to the selected genome after filtering of the BLAT exons. The "*Evaluation*" output classifies the cDNAs according to their quality and the presence or lack of the features described above. It provides the following fields: contamination, number of mismatches in the 5' and 3'end, mapping classification, exon number, splice site number, score and type, polyA signal number type and score, repeats, repeats in the 3' end. "*VEGA annotation*" and "*ENSEMBL annotation*" outputs contain detailed annotation of genes and transcripts in VEGA/ENSEMBL overlapping the analyzed cDNAs. This file includes a quality score for each overlapping gene and transcript that is based on the number of shared positions over the total length of the overlapping annotated gene/transcript. The table also contains gene identifiers, begin and end of mapping positions, chromosome number, number of exons, and information on shared and missed exons. "*VEGA detailed annotation*" and "*ENSEMBL detailed annotation*" outputs provide further details at the individual exon level, plus positional differences between annotated VEGA/ENSEMBL exons and exons of the query cDNA. The "*Polyasignal*" output contains the results of the search for polyA signals and polyA tails in the cDNAs using the selected regular expressions. The final output of CAFTAN is an "*XML*" file that contains all information from all outputs obtained in the process. This XML file can be directly integrated in user databases and used for further analysis with other programs.

Currently we are working on the development of supervised learning algorithms for the improvement of cDNA quality prediction, especially for single exon cDNAs. In the future CAFTAN will be additionally extended towards the classification of transcripts that are products of exon skipping events, alternative usage of Poly (A) sites, and the presence of alternative promoters and transcription start sites (TSS).

The results described here were obtained with the default parameters established for CAFTAN and optimized for mammals. One of the advantages of CAFTAN is that it allows the adjustment of parameters to adapt it to any organism. The parameters can easily be changed in the configuration file (Config.pm). The polyAsignal program can be configured for other genome preferences, and the rules to be taken into account can be modified for different genomes. New organism's genomic databases can be used as soon as they become available by adding them to the configuration file. We think that it is a very useful program for the annotation and research of unfinished genomes. The use of CAFTAN as a first filtering step should be followed by an ORF analysis like described by Takeda et al. [51]. We further plan to extend the algorithm to the analysis of ESTs as well.

## Conclusion

We have developed a new high-throughput sequence analysis tool, CAFTAN [see Additional file 1], which performs a fast and reliable quality prediction of cDNAs. Several thousand cDNAs can be analyzed in a short time, giving the curator/scientist a first quick overview about the quality and the already existing annotation of a set of cDNAs. CAFTAN does not substitute the manual expert curation and further detailed ORF analysis; however it supports the rejection of low quality cDNAs and the selection of likely novel splice variants, and/or completely novel transcripts. The successful exploitation of the large number of available cDNA sequences and the respective clones in functional genomics experiments necessitates the fast distinction between noise and those cDNAs that are valid for further detailed and manual annotation.

## Limitations

- Existing databases in ENSEMBL

- The need of a BLAT mapping output.

- The performance of CAFTAN depends on the number of input cDNAs and on the overall machine load. Analysis of the 4,000 cDNAs from the VEGA set took 21.89 seconds on a six processor SUN Enterprise. The remote use of the MSQL ENSEMBL database could be slowed down when the ENSEMBL server is extensively accessed, favoring a local implementation.

## Availability

Operating system(s): Linux, Unix

Programming language: Perl5

Requirements:

- For the download distribution it is necessary to install ENSEMBL package, bioperl-2.1 or higher, and the Perl DBI Package prior to the installation of CAFTAN.

- Use of the web version requires a HUSAR account at [52].

License:

Restrictions to use by non-academics: license needed

## Authors' contributions

V.K conceived the initial idea. C.V, V.K and KH.G developed and designed the project. C.V and P.E designed and coded the program Polyasignal, and designed CAFTAN classes as well as the downloadable module. C.V. developed the tests, coded the program, created the test sets, and drafted the manuscript. A. H-W created the XML schema for CAFTAN. A.P, S.W and S.S participated in coordination; curated manually the "bad" cDNAs set and helped to draft the manuscript. All authors read and approved the final version of the manuscript.

## Supplementary Material

Additional file 4 Table of DKFZ annotated sequences Click here for file

Additional file 5 Hand annotation of the DKFZ cDNAs sequences Click here for file

Additional file 2 polyasignal executable for EMBOSS Click here for file

Additional file 3 polyasignal acd file for EMBOSS Click here for file

Additional File 1 CPAN distribution of the CAFTAN software Click here for file
