# Supplementary material for: CAFTAN: a tool for fast mapping, and quality assessment of cDNAs
Source: BMC Bioinformatics. 2006 Oct 25;7:473. doi: 10.1186/1471-2105-7-473 (PMC1636072; doi:10.1186/1471-2105-7-473)
Supplement: Additional file 4 — Table of DKFZ annotated sequences [file 1471-2105-7-473-S4.doc]

Explanatory Table to the terms used by the curators in the supplementary Table

| Table key | Content |
| --- | --- |
| Acc | Genebank accession number |
| **qSize** | cDNA length |
| **nopA_qSize** | Length of the cDNA without polyA tail |
| **Exons_nr** | Number of exons in the cDNA |
| **DKFZ_ID** | DKFZ identification number |
| **5delta** | It is the cDNA start mapping position in the genome is < 28 bp |
| **3delta** | It is the difference between the length of the cDNA and the last position mapped in the 3' end of the cDNA without poly (A) tail |
| **Intdelta** | It is the number of mismatches in the cDNA exons taking into account the cDNA length |
| **% Unmapped** | Total mapping |
| **Mapping sign** | Quality of the mapping:  *Mapped, 5UTR unmapped, 3UTR unmapped, Internal unmapped, more than one significant hit (2hits, 3hits, 4hits ... nhits)* |
| **CpG-island** | Presence (CpG) or absence (noCpG) of a CpG island |
| **100bp -5' flank genomic DNA repeat** | Presence of a repeat in the 100bp downstream of the cDNA 5’ end |
| **100bp -5' flank genomic DNA repeat familiy** | Type of repeat in the 100bp downstream of the cDNA 5’ end |
| **50bp -5' flank genomic DNA repeat** | Presence of a repeat in the 50bp downstream of the cDNA 5’ end |
| **50bp -5' flank genomic DNA repeat familiy** | Type of repeat in the 50bp downstream of the cDNA 5’ end |
| **100bp –3 'flank genomic DNA repeat** | Presence of a repeat in the 100bp upstream of the cDNA 3’ end |
| **100bp -3' flank genomic DNA repeat familiy** | Type of repeat in the 100bp upstream of the cDNA 3’ end |
| **50bp -3' flank genomic DNA repeat** | Presence of a repeat in the 50bp upstream of the cDNA 3’ end |
| **50bp -3' flank genomic DNA repeat familiy** | Type of repeat in the 50bp upstream of the cDNA 3’ end |
| **Genomic polyA** | If there is more than 80% A’s in a 20bp window in the genome, taking into account the 5 last b.p. from the last exon + 15bp after. |
| **polyA tail** | Presence and polyA tail in the cDNA (yes or no) |
| **polyA signal type** | Type of polyA signal in the cDNA (yes or no) |
| **polyA-signal** | Presence of polyA signal in the cDNA; nopAsignal: no poly A signal detected |
| **SpliceSites** | Types of splice sites  *Unknown: at least one Unknown splice sites* Antisense: at least one antisense Ss, no Unknown *No-canonical: Non_canonical or U12 Ss*  *Canonical: All splice sites are canonical*  *NoSS: no splice sites, single exons* |
| Contamination | Presence and type of contamination  *ACONT: if there is a genomic polyA*  *RCONT: presence of complex repeats contamination in the last exon or in the 3’ end* ICONT: internal contamination *NORM: No Contamination*: |
| **Quality** | Tag given to the cDNA by the curator:  *Unmapped*  *Chimeric*  *Deleted_multi_exon*  *Deleted_single_exon* Good_multi_exon *Good_single_exon* Questionable_multi_exonQuestionable_single_exon *Bad_antisense*  *Bad_cont* |
